# Purification, Biochemical and Kinetic Characterization of a Novel Alkaline *sn*-1,3-Regioselective Triacylglycerol Lipase from *Penicillium* *crustosum* Thom Strain P22 Isolated from Moroccan Olive Mill Wastewater

**DOI:** 10.3390/ijms231911920

**Published:** 2022-10-07

**Authors:** Ismail Hasnaoui, Ahlem Dab, Sondes Mechri, Houssam Abouloifa, Ennouamane Saalaoui, Bassem Jaouadi, Alexandre Noiriel, Abdeslam Asehraou, Abdelkarim Abousalham

**Affiliations:** 1 Univ Lyon, Université Lyon 1, Institut de Chimie et de Biochimie Moléculaires et Supramoléculaires (ICBMS), UMR 5246 CNRS, Génie Enzymatique, Membranes Biomimétiques et Assemblages Supramoléculaires (GEMBAS), Bât Raulin, 43 Bd du 11 Novembre 1918, CEDEX, F-69622 Villeurbanne, France; 2 Laboratoire de Bioressources, Biotechnologie, Ethnopharmacologie et Santé (LBBES), Faculté des Sciences d’Oujda (FSO), Université Mohammed Premier (UMP), Bd Mohamed VI, BP 717, Oujda 60000, Morocco; 3 Laboratoire de Biotechnologie Microbienne et d’Ingénierie des Enzymes (LBMIE), Centre de Biotechnologie de Sfax (CBS), Université de Sfax, Route de Sidi Mansour Km 6, BP 1177, Sfax 3018, Tunisia; 4 Research Unit of Microbiology, Biomolecules and Biotechnology, Laboratory of Chemistry Physics and Biotechnology of Molecules and Materials, Faculty of Sciences and Techniques Mohammedia, Hassan II University of Casablanca, Mohammedia 28806, Morocco

**Keywords:** detergent formulation, lipase, orlistat, olive oil, olive mill wastewater, organic solvents, *Penicillium*, regioselectivity

## Abstract

A novel extracellular lipase from a filamentous fungus Ascomycota strain, P22, was isolated from olive mill wastewater, then purified and characterized. This strain was identified as *Penicillium crustosum* Thom based on sequencing analyses. *Penicillium* *crustosum* Thom strain P22 lipase (PCrL) was purified 63-fold to homogeneity using ammonium sulfate precipitation and chromatography on a Q-Sepharose Fast Flow column, with a total yield of 34%. The purified PCrL had a molecular mass of 28 kDa, estimated by SDS-PAGE. The 20 NH_2_-terminal amino-acid residues showed a high degree of homology with those of other *Penicillium* lipases. The specific activity of PCrL at pH 9 and 37 °C were found to be 5000 and 10,000 U/mg on olive oil and trioctanoin emulsions, respectively. PCrL exhibited clear regioselectivity toward the *sn*-1 position of the surface-coated triglycerides which were esterified with α-eleostearic acid at the *sn*-1/3 position. PCrL was completely inhibited by 53 µM of Orlistat, 5 mM of phenylmethylsulfonylfluoride, and 2 mM of diiodopropyl fluorophosphate, suggesting that it belonged to the serine lipase family. PCrL showed high activity and stability in the presence of water-immiscible organic solvents, surfactant, and oxidizing agents, and showed considerable compatibility with commercial laundry detergents. Washing performance analysis revealed that it could effectively remove oil stains. Hence, PCrL has several attractive properties that make it a promising potential candidate for detergent formulations.

## 1. Introduction

Olive oil (OO) production has increased significantly worldwide in recent years [1]. In terms of both health and agriculture, the production of OO is a vital industry for several Mediterranean countries. However, such production has led to a much larger number of inedible components. In fact, in the OO extraction process, 20% of oil is acquired, while 80% is considered as olive mill wastewater (OMW) [2,3,4]. The valorization of OMW is actually a challenging opportunity for the viable and competitive development of the OO industry. As a bioprocess, modern scientific explorations have shown that OMW contains useful lipase-producing microorganisms, and these can be exploited biotechnologically [5]. Lipases (triacylglycerol acylhydrolases, EC 3.1.1.3) are carboxylester hydrolases that catalyze the hydrolysis of long-chain acylglycerols at the lipid–water interface [6,7]. In addition, they catalyze the reverse reactions, such as esterification, interesterification, and transesterification, under non-aqueous conditions [8,9]. Owing to their broad spectrum of catalytic reactions, lipases are used in several industrial applications, such as detergent formulations, biodiesel synthesis, the production of pharmaceutical and cosmetic esters, food, and wastewater treatment [10,11,12]. Lipases are ubiquitous in nature and can be produced by a variety of plants, animals, and microorganisms [13]. However, microbial lipases are the most commercially useful for their indisputable role among biocatalysts [14]. Fungi are the preferred source, as they generally produce extracellular enzymes, facilitating the recovery of the enzyme from the fermentation broth [15]. Lipase-producing fungi are especially found in waste from the vegetable oil and dairy industries, oil-contaminated soils, and spoiled food [16].

Among fungi, the genus *Penicillium* contains many lipases that have attracted considerable industrial interest due to their high activity, stability, and broad substrate-specificity [17]. For example, *Penicillium* lipases have been used in the production of monoacylglycerides [18], in cheese ripening, and in the production of flavors in dairy products [19]. Their main application remains as additives for laundry detergents [20,21]. In addition, several *Penicillium* lipases, such as lipase G Amano 50^®^ from *Penicillium camembertii*, lipase R Amano^®^ from *Penicillium roqueforti* (Amano Enzyme Inc., Nagoya, Japan), and lipase LVK^®^ from *Penicillium expansum* (Shenzhen Leveking Bioengineering Co., Ltd., Shenzhen, China), are commercially available [22]. Industrial processes generally require enzymes with a high catalytic potential accompanied by tolerance to many environmental perturbations, such as pH, temperature, metal ions, and inhibitors [23]. However, the high production costs of these biocatalysts often limit their industrial use. The search for new sources of lipases is justified by the many possibilities for future applications requiring not only enzyme–substrate specificity, but also process stability, such as a broad pH tolerance and thermal stability of the biocatalyst. Most microbial lipases have been found to be active at alkaline pH (7–9), with a few exceptions [24,25,26].

Thus, the current study focuses on the production, purification, and biochemical characterization of a new inducible extracellular lipase from *Penicillium crustosum* Thom strain P22 (PCrL) isolated from the OMW of traditional Moroccan oil mills. The characterization of the PCrL and comparison with some commercial and previously analyzed lipases have been methodically elaborated. The stability of the PCrL was assessed in the presence of several organic solvents and detergents. Finally, the destaining potential of the purified PCrL lipase was investigated. The characterization of the catalytic lipase activity was performed to meet the requirements of biotechnological applications and for the valorization of OMW according to biotechnological criteria.

## 2. Results

### 2.1. Fungal Isolation and Identification

Thirty fungal isolates, all morphologically different, were isolated from Moroccan OMW. The identification of the isolated P22 strain was based on both morphological and molecular methods. Morphological observation of strain P22 typified it as a filamentous fungus, characterized by septate hyphae, conidiophore, and conidial structures exhibiting vesicles and sterigmata. These characteristics are similar to those of *Penicillium* species.

**Figure 1:**
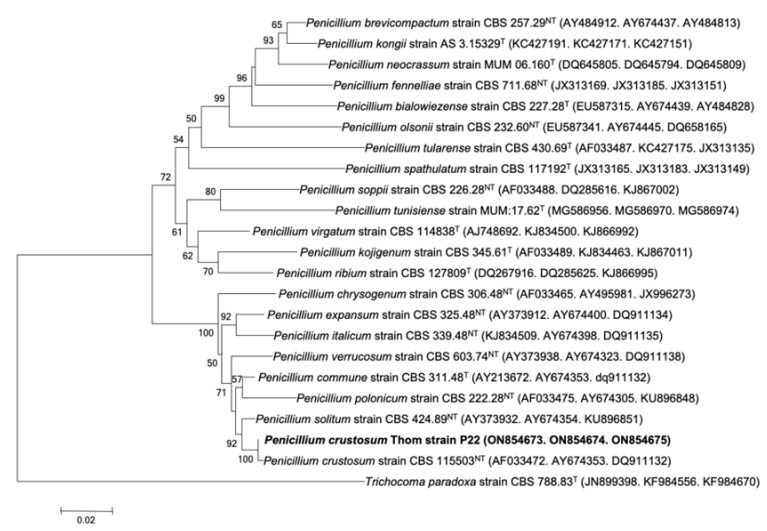
Strain P22 identification. Phylogenetic tree based on the combined phylogeny for *ITS*1-5.8S-*ITS*2, *benA*, and *cmd* genes sequences showing the position of *Penicillium crustosum* Thom strain P22 within the cluster comprising *Penicillium* species. The sequences of *Trichocoma paradoxa* strain CBS 788.83 (GenBank accession no.: JN899398) were chosen as an outgroup. Bar, 0.02 substitutions per nucleotide position. Numbers at nodes (>50%) indicate support for the internal branches within the tree obtained by bootstrap analysis (percentages of 100 bootstraps). GeneBank accession numbers are presented in parentheses. T, ex-type strain; HT, holotype strain; NT, neotype strain.

In order to establish additional details for the identification of the P22 isolate, a molecular approach was followed. Sequencing of three genes (*ITS*, *benA*, and *cmd*) was performed. The BLAST results showed that it *Penicillium crustosum* (sequence identity ≥ 99%) had the closest alignment, indicating a possible connection with *Penicillium* section *Fasciculata*. To confirm its phylogenetic placement within *Penicillium*, combined sequences (*ITS*, *benA*, and *cmd*) were aligned against those of the type strains belonging to the section *Fasciculata* and the closely related sections *Penicillium*, *Ramosa*, and *Brevicompacta*. In the resulting phylogenetic tree (Figure 1), the unidentified isolate P22 was placed in a monophyletic clade composed exclusively of section *Fasciculata* strains and supported by 71% of the bootstrap samples. Accordingly, the isolate P22 was classified as *Penicillium* section *Fasciculata* clustering with *Penicillium crustosum* strain CBS 115503^NT^ and *Penicillium solitum* strain CBS 424.89^NT^ (supported by 92% of the bootstrap samples). The sequence analyses of the *ITS*1-5.8S-*ITS*2 region (879 bp), as well as the *benA* (480 bp) and *cmd* (329 bp) genes, indicates the presence of three isogenic (ON854673, ON854674, and ON854675, respectively) isolates belonging to the species *crustosum* of the genus *Penicillium* section *Fasciculata*, described and named *Penicillium crustosum* Thom strain P22.

### 2.2. Lipase Production

**Figure 2:**
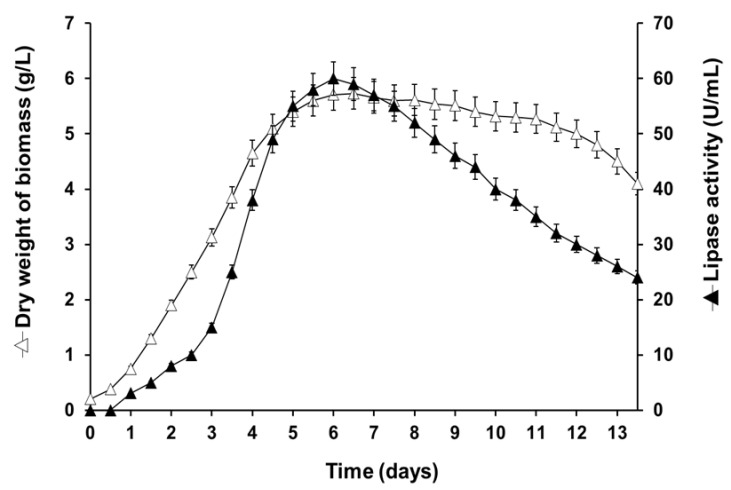
Time course of the production of PCrL from strain P22 after cultivation in liquid medium. The cultures were carried out in 250 mL Erlenmeyer flasks as described in Section 4 (Section 4.6). Lipase activity was measured using TC8 as the substrate. Each data point (mean ± SD) is the result of triplicate experiments.

The fungal strain P22, isolated from Moroccan OMW, exhibited lipase activity after 5 days of incubation at 25 °C on agar plates containing OO and rhodamine B. Lipase production was confirmed by submerged culture in a liquid medium. The supernatant showed lipase activity from the 2nd day of culture, using glyceryl trioctanoate (TC8) as the substrate, reaching a maximum activity of 60 U/mL after 6 days of culturing (Figure 2). The maximum activities of 18 and 40 U/mL on OO emulsion and glyceryl tributyrate (TC4), respectively, were determined using the pH-Stat technique under standard assay conditions.

### 2.3. Purification of PCrL

**Figure 3:**
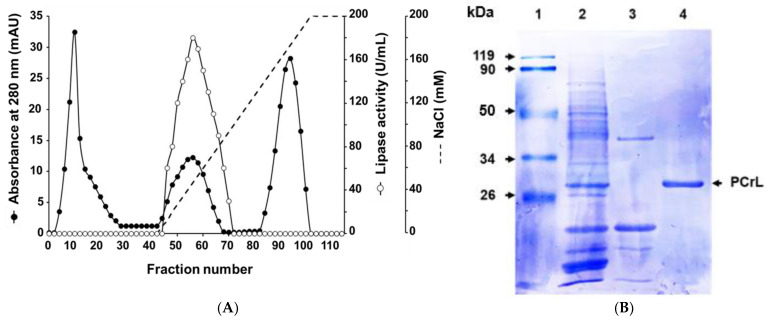
Purification of PCrL. (**A**) Chromatogram profile of PCrL purification on HiTrap™ Q-Sepharose FF column. Adsorbed proteins were eluted with a linear NaCl gradient of 0–200 mM NaCl in buffer A. PCrL activity was measured, as described in Section 4 (Section 4.5), using TC8 as the substrate. (**B**) SDS-PAGE (12% acrylamide) analysis of eluted proteins. Lane 1, molecular mass marker; lane 2, resuspended pellets after ammonium sulfate (70%) precipitation; lane 3, non-retained proteins; lane 4, pooled active fractions from HiTrap™ Q-Sepharose FF column.

PCrL was purified to homogeneity from the culture supernatant as described in Section 4 (Section 4.8). Purification was performed in one chromatographic step using the HiTrap™ Q-Sepharose Fast Flow (FF) column by applying a linear gradient of NaCl from 0 to 200 mM in buffer. The elution profile showed a single major peak of PCrL activity at approximately 50 mM NaCl (Figure 3A). The results of the purification procedure are summarized in Table 1. The enzymatic purity was estimated to be 62.5-fold higher than that of the crude extract, with an overall yield of about 34%. From 160 mL of 6-day-old culture medium, about 0.26 mg of pure PCrL was obtained with a specific activity of 10,000 U/mg when using TC8 as the substrate.

To analyze the homogeneity and molecular mass of the purified PCrL, polyacrylamide gel (12%) electrophoresis in the presence of sodium dodecyl sulfate (SDS-PAGE) was performed. Highly purified PCrL, devoid of any detectable contaminants, yielded a single protein band at a position corresponding to a molecular mass of 28 kDa, as assessed by SDS-PAGE (Figure 3B).

The NH_2_-terminal amino-acid sequencing of the pure PCrL, allowing the identification of 20 residues, showed uniformity, indicating that it was isolated in a pure form (Table 2). This NH_2_-terminal sequence exhibited a high degree of homology (70–90%) with lipases of the same previously characterized genus (Table 2).

### 2.4. Biochemical Characterization of PCrL

#### 2.4.1. Effects of Temperature on PCrL Activity and Stability

**Figure 4:**
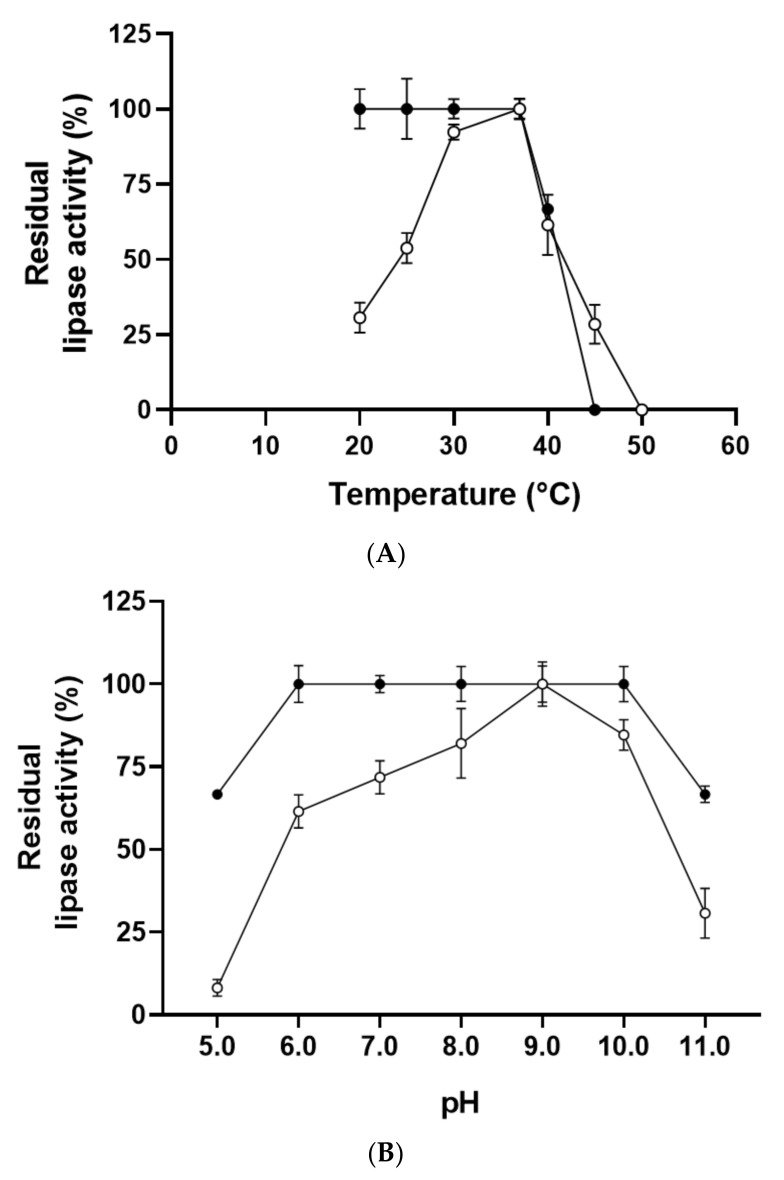
Effects of temperature and pH on the activity and stability of the purified PCrL. (**A**) The temperature profile (filled circle) was determined by assaying enzyme activity at various temperatures and pH 9. The activity of the enzyme at 37 °C was taken as 100%. The temperature stability (empty circle) was determined by incubating the PCrL at different temperatures. The residual enzyme activity was measured at 37 °C and pH 9 using TC8 as the substrate. Each point represents the mean of three independent experiments. (**B**) The pH profile (filled circle) was determined in different buffers by varying the pH values from 5 to 11. The maximum activity obtained at pH 9 was considered as 100%. The pH stability (empty circle) of the PCrL was determined by incubating the enzyme at different pH values, ranging from 5 to 11, for 1 h at 4 °C, and the residual activity was measured at pH 9 and 37 °C using TC8 as the substrate. Each point represents the mean of three independent experiments.

Lipase activity was evaluated from 20 to 50 °C (Figure 4A), and PCrL showed maximum activity at 37 °C. At 40 °C, the enzyme exhibited 60% of its maximum activity. After 60 min of incubation of PCrL at various temperatures, the PCrL was shown to be stable between 20 and 37 °C, but the thermal stability of the enzyme declined at 40 °C, retaining approximately 60% of its activity (Figure 4A).

#### 2.4.2. Effects of pH on PCrL Activity and Stability

The purified PCrL was found to be highly active between pH 8 and pH 10, with an optimal pH of 9; therefore, it can be considered as an alkaline enzyme (Figure 4B). Furthermore, the activity found between pH 6 and pH 8 represents approximately 60 to 80% of that observed at the optimum pH (Figure 4B).

The pH stability of PCrL was assessed by pre-incubating the enzyme over a wide pH range of 5–11 for 1 h at 37 °C. As shown in Figure 4B, PCrL exhibited exceptional stability at pH values ranging from 6 to 9, while it could even retain about 70% residual activity at pH 5 and pH 11.

#### 2.4.3. Effect of Inhibitors, Metal Ions, and Bile Salts on the PCrL Activity

**Figure 5:**
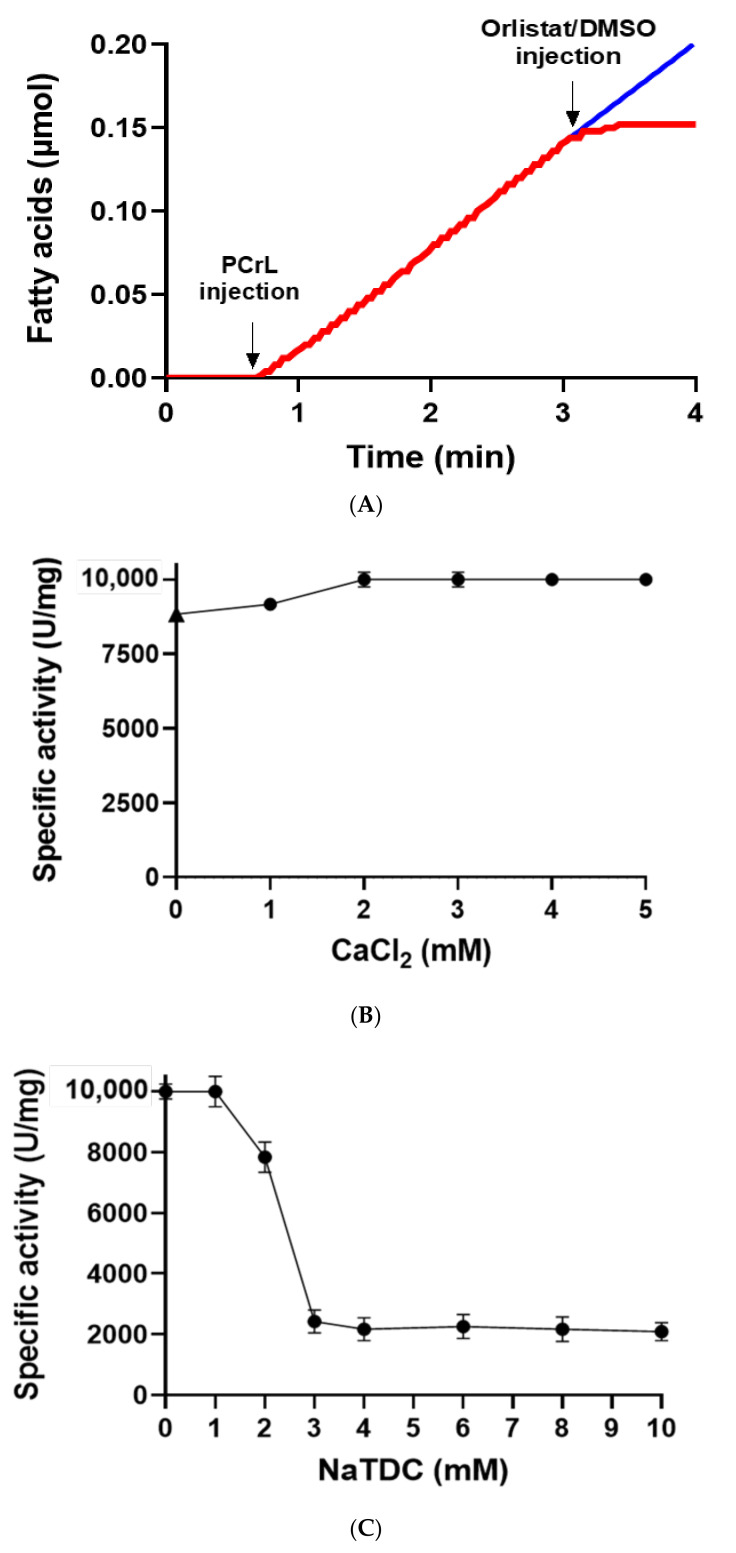
Influence of Orlistat, calcium, and sodium taurodeoxycholate on the PCrL activity. (**A**) Effect of Orlistat on PCrL activity. After PCrL injection, DMSO (blue line) or Orlistat in DMSO (53 µM final concentration, red line) were injected into the reaction medium during lipolysis 3 min after starting the reaction. Curves are representative of three independent experiments. (**B**) Effect of the concentration of Ca^2+^ on PCrL activity. Enzyme activity was measured at increasing concentrations of Ca^2+^ using TC8 as the substrate at 37 °C and pH 9. The triangle indicates the lipase activity measured in the absence of Ca^2+^ and in the presence of 10 mM EDTA. (**C**) Effect of an increasing concentration of bile salt (NaTDC) on lipase activity (filled circle) in the presence of 2 mM of Ca^2+^ using TC8 as the substrate at 37 °C and pH 9.

To verify whether PCrL is a serine lipase, we tested the effect of Orlistat, a known potent digestive lipase inhibitor that covalently binds to lipase active site serine [27], on its activity during lipolysis by injecting dimethylsulfoxide (DMSO) alone (Figure 5A, blue curve) or Orlistat (53 µM, final concentration, red curve) at 3 min. As can be seen from Figure 5A, PCrL was completely inhibited by Orlistat. Additionally, phenylmethanesulfonyl fluoride (PMSF) and diiodopropyl fluorophosphate (DFP), other-serine modifying reagents, were shown to completely inhibit PCrL’s activity (Table 3).

Moreover, PCrL’s activity was shown not to be significantly affected by treatment with thiol reagents (5,5′-Dithiobis 2 nitrobenzoic acid (DTNB), N-ethylmalemide (NEM), iodoacetamide, and phenylarsine oxide (PAO)) (Table 3), suggesting that cysteine residues at or near its active site were not essential for PCrL function. Furthermore, PCrL activity was slightly inhibited by chelating reagents, such as ethylene-diaminetetraacetic acid (EDTA) and ethylene glycol-bis(2-aminoethylether)-*N*,*N*,*N*′,*N*′-tetraacetic acid (EGTA) (Table 3), which seems to confirm that metal cofactors may not be essential for activity. A marginal effect on PCrL activity was detected with reducing agents, such as β-mercaptoethanol (β-ME) or dl-dithiothreitol (dl-DTT), which may mean that disulfide bonds were not crucial for preserving the active conformation of PCrL.

The effect of some divalent ions on the activity of PCrL was also tested. Metal ions could be important in maintaining three-dimensional structure and enzyme function. An increase in enzyme activity was observed following incubation with Ca^2+^ or Mn^2+^ (147 or 146%, respectively), compared with the control (Table 3). No significant effect on PCrL activity was observed with Ba^2+^ and Mg^2+^. However, Fe^2+^ and Co^2+^ decreased the PCrL activity by over 30 and 60%, respectively (Table 3). In addition, the heavy metal ions Cu^2+^, Ni^2+^, and Zn^2+^ inhibited the enzyme activity by 77, 84, and 91%, respectively (Table 3).

Concerning the effect of Ca^2+^, Figure 5B shows that Ca^2+^ seemed not to be necessary for triggering the activity of PCrL, which could be detected (8700 U/mg) even in the absence of Ca^2+^ and in the presence of 10 mM EDTA as a chelator. The specific activity of PCrL reached its maximum (10,000 U/mg) at 2 mM of CaCl_2_ without chelators, suggesting that PCrL was partially calcium-dependent.

In order to determine if the purified PCrL was able to hydrolyze TGs in the presence of surface active agents, such as bile salts, the hydrolysis rate of TC8 was measured in the presence of increasing concentrations of sodium taurodeoxycholic acid (NaTDC). As can be seen from Figure 5C, PCrL reached its maximum specific activity (10,000 U/mg) in the absence of detergent, and this activity persisted for up to 1 mM of NaTDC. However, beyond 1 mM of NaTDC, the activity of the enzyme decreased rapidly to stabilize at 20% of the residual activity at NaTDC concentrations greater than or equal to 3 mM (Figure 5C).

### 2.5. Substrate Specificity

**Figure 6:**
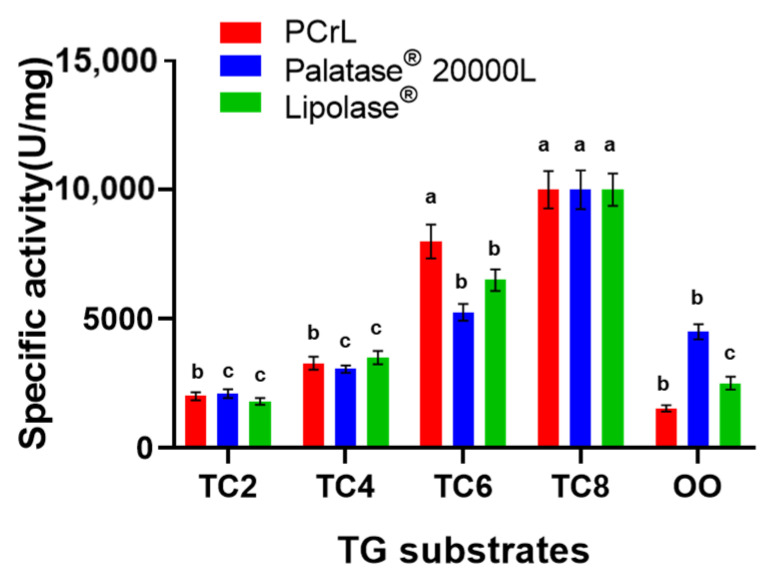
Substrate specificity of PCrL. Lipase activity was measured using TC2, TC4, TC6, TC8, or OO emulsion as the substrate, as described in Section 4 (Section 4.9.3). ^a–c^ Means in indicator enzymes with different lowercase letters differed significantly (*p* < 0.05).

The substrate specificity of the PCrL was determined using triglycerides (TGs) of varying acyl chain lengths and compared with that of Palatase^®^ 20000 L and Lipolase^®^ (Figure 6). PCrL was able to hydrolyze TGs with acyl chains ranging from 2 to 18 carbons long, with an optimal activity for TC8 followed by glyceryl trihexanoate (TC6), but it showed very low activity toward TGs with short-chain fatty acids (shorter than 4 carbons). For the sake of comparison, Palatase^®^ 20000 L and Lipolase^®^ were shown to have almost the same substrate specificity profile as PCrL under the same experimental conditions (Figure 6).

**Figure 7:**
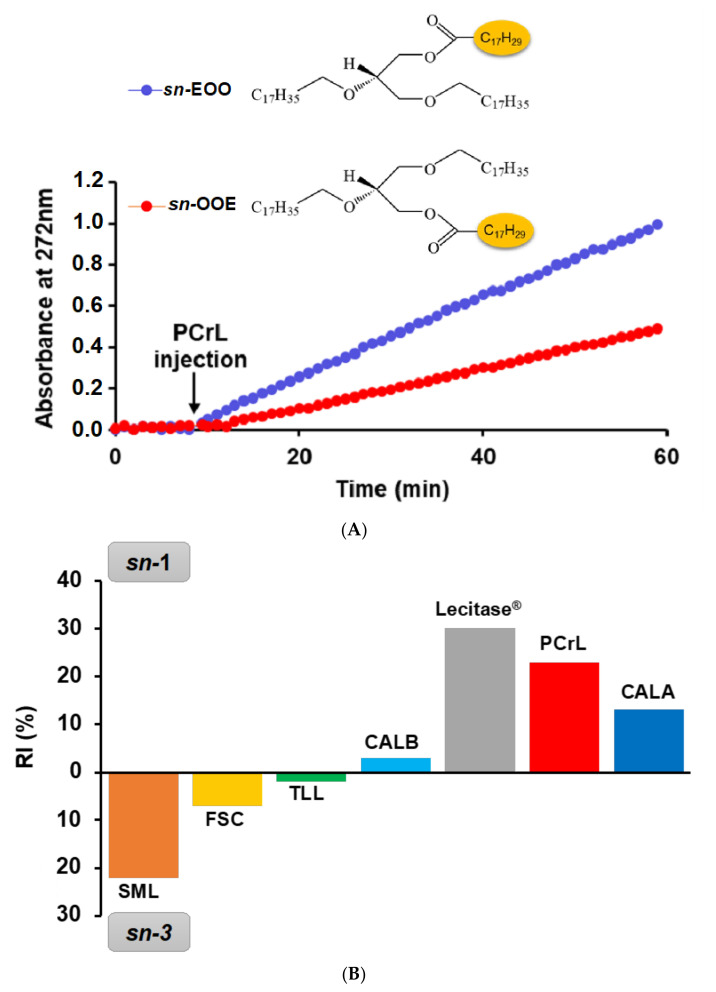
Regioselectivity of PCrL toward synthetic TGs. (**A**) Kinetics of surface-coated *sn*-EOO or *sn*-OOE hydrolysis by PCrL. The variation in absorbance at 272 nm was recorded for 10 min to obtain the baseline, then for 50 min after the injection of PCrL (0.65 µg per well). The kinetic recordings shown here are typical of those obtained in three independent experiments. (**B**) RI% of PCrL using surface-coated *sn*-EOO or *sn*-OOE compared with those of SML, FSC, TLL, Lecitase, CALA, and CALB reported in our previous studies [28,29].

To investigate the regioselectivity of PCrL for the *sn*-1 or the *sn*-3 positions of the TG substrate, the rate of hydrolysis of surface-coated 1-α-eleostearoyl-2,3-octadecyl-*sn*-glycerol (*sn*-EOO) or 1,2-octadecyl-3-α-eleostearoyl-*sn*-glycerol (*sn*-OOE) was determined (Figure 7A). An increase in absorbance at 272 nm was observed after PCrL had been injected. This increase was due to the release of α-eleostearic acid from its adsorbed state to its soluble state following the action of PCrL on *sn*-EOO (Figure 7A, blue curve) or *sn*-OOE (Figure 7A, red curve). It is worth noting that the kinetic slope of PCrL was higher when using *sn*-EOO than that when using *sn*-OOE, indicating a clear regioselectivity of PCrL for the *sn*-1 position of the TG substrate. The specific activities were calculated to be 74 ± 2 and 45 ± 1.5 U/mg using *sn*-EOO and *sn*-OOE, respectively.

To quantitatively assess the regioselectivity of PCrL toward a given TG substrate, the regioselectivity index (RI) was calculated. PCrL hydrolyzes both positions (*sn*-1 and *sn*-3) of the TG, showing a clear regioselectivity toward the *sn*-1 position with an RI of 23% (Figure 7B). The RI value of PCrL was compared with the value previously determined for other microbial lipases [28] (Figure 7B). Based on the RI, 1,3-regioselective lipases could be classified into one of three major groups. The first group, with no significant regioselectivity, included *Thermomyces lanuginosus* lipase (TLL) and *Candida antarctica* B lipase (CALB), with RI of 2 and 3%, respectively [28] (Figure 7B); the second group, showing a significant regioselectivity for the *sn*-3 position, such as the lipase from *Serratia sp*. W3 (SML) [29] and *Fusarium solani* cutinase (FSC), had RI of 22 and 7%, respectively [28] (Figure 7B); and the third group of lipases showing a clear regioselectivity for the *sn*-1 position of the TGs, including *Candida antarctica* A lipase (CALA) and Lecitase^®^, had RI of 13 and 30%, respectively [28]. PCrL displayed a clear regioselectivity for the *sn*-1 position, with an RI value of 23% (Figure 7B); thus, it would be classified within the third group with CALA and Lecitase^®^. These features make PCrL very attractive for application in transesterification reactions involving the *sn*-1 position.

### 2.6. Enzymatic Performance of the Purified PCrL

#### 2.6.1. Effect of Organic Solvents on Enzyme Stability

**Figure 8:**
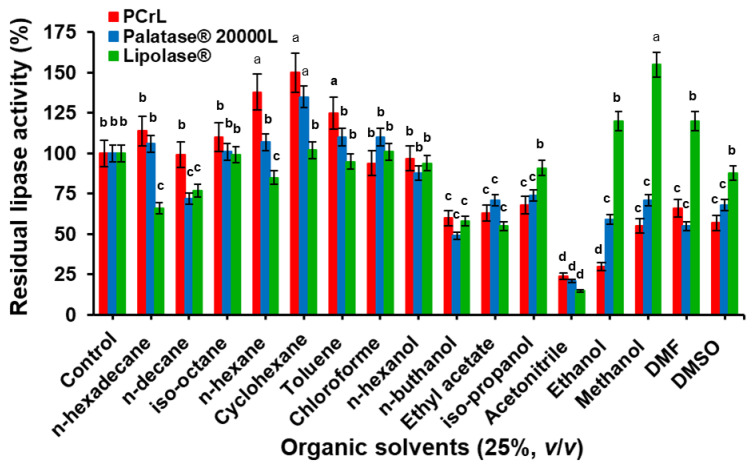
Effect of organic solvents on the activity and stability of PCrL, Palatase^®^ 20000 L, and Lipolase^®^. The effect of organic solvents was determined by incubating each enzyme with the solvent (25% (*v*/*v*) final concentration) for 24 h. The residual lipase activities were determined under the same conditions, using TC8 as the substrate at 37 °C and pH 9, as described in the Materials and Methods, and then expressed as a percentage of the activity level in the absence of organic solvents. The activity of the enzyme without any organic solvent was taken as 100%. Each point represents the mean of three independent experiments. Vertical bars indicate standard error of the mean (*n* = 3). ^a–d^ Means in indicator enzymes with different lowercase letters differed significantly (*p* < 0.05).

The effect of organic solvents on lipase stability was determined for PCrL and, as a comparison, for the commercially available lipases Lipolase^®^ and Palatase^®^ 20000 L (Figure 8). After 24 h of incubation, at a final concentration of 25% (*v*/*v*) of water-immiscible organic solvents (Log *P* > 1.8), PCrL showed good stability, with a residual activity of 114, 98, 110, 138, 150, 125, 94, and 97% in the presence of *n*-hexadecane, *n*-decane, *iso*-octane, *n*-hexane, cyclohexane, toluene, chloroform, and *n*-hexanol, respectively, compared with that of the control without solvent (100%) (Figure 8). Almost the same stability profile in these organic solvents was obtained with Palatase^®^ 20000 L, while Lipolase^®^ was found to be less stable in these water-immiscible organic solvents, although with a residual activity greater than 60% (Figure 8).

In contrast, the exposure of PCrL to water-miscible (polar) organic solvents showed poor enzyme stability (Figure 8). In the presence of DMF, DMSO, or methanol, PCrL conserved 66, 57, or 55% of its activity, while it lost about 70 or 76% of its activity when incubated with ethanol or acetonitrile, respectively (Figure 8). Palatase^®^ 20000 L, under the same experimental conditions, retained 68, 55, 71, 59, and 21% of its initial activity in the presence of DMSO, DMF, ethanol, and acetonitrile, respectively (Figure 8). It is worth noting that, with the exception of acetonitrile, the other water-miscible organic solvents ethanol, methanol, DMF, and DMSO all had a positive effect on the stability of Lipolase^®^, with residual activities of 120, 155, 120, and 88%, respectively (Figure 8).

#### 2.6.2. Influence of Some Laundry Detergent Constituents on PcrL Stability

The activity of PCrL, as well as that of the commercial lipases Palatase^®^ 20000 L and Lipolase^®^ for comparison purposes, was tested after incubation for 1 h with some laboratory-available (Table 4) or commercialized (Table 5) detergent additives. Residual PCrL activity of 152, 154, and 182% was observed in the presence of SAFOL 23E7, Dehydol^®^ LT 7, and SURFAC^®^ LM 30, as non-ionic surfactants, at 0.5% (*v*/*v*), respectively (Table 5). Moreover, PCrL exhibited considerable stability against some strong anionic surfactants. Its stability in the presence of 2% (*v*/*v*) Galaxy 110, 1% (*v*/*v*) Galaxy CAPB plus, and 2% (*w*/*v*) SDS increased by 149, 152% (Table 5), and 185% (Table 4), respectively.

In the presence of 0.5% (*v*/*v*) SAFOL 23E7, 2% (*v*/*v*) Galaxy 110, and 2% (*w*/*v*) SDS, the Palatase^®^ 20000 L retained 145, 150% (Table 5), and 90% (Table 4) of its initial activity, respectively, whilst Lipolase^®^ retained, respectively, 142, 125% (Table 5), and 85% (Table 4) of its initial activity. For the oxidizing agents, the stability of PCrL in the presence of H_2_O_2_ at 5 and 10% (*v*/*v*) increased by 145 and 104%, respectively (Table 4). Moreover, the enzymatic activity in the presence of anti-redeposition agents was also very interesting. PCrL exhibited 141, 142, 146, and 149% of its initial activity with 0.5% (*w*/*v*) tetraacetylethylenediamine (TAED), 1% (*w*/*v*) sodium carboxymethylcellulose (Na_2_ CMC), 100 mM Na_2_ CO_3_, and 0.2% (*w*/*v*) sodium tripolyphosphate (STPP), respectively (Table 4). The PCrL activity was stable, with a residual activity of 108–170% in other commercialized detergent additives, particularly (*v*/*v*) 0.5% Formol, 0.5% Tinopal^®^ CBS-X, 0.2% EDTA, 0.5% Perfume I Class, and 1% Perfume II Class (Table 5). In contrast, in the presence of 5% (*v*/*v*) H_2_O_2_, 0.5% (*w*/*v*) TAED, and 0.5% Formol, Palatase^®^ 20000 L retained 61, 125% (Table 4), and 152% (Table 5) of its initial activity, respectively, and Lipolase^®^ retained 80, 115% (Table 4), and 135% (Table 5) of its initial activity, respectively.

These results clearly indicate that PCrL exhibited at least the same stability, or even better stability, than Palatase^®^ 20000 L and Lipolase^®^ with most detergent ingredients, providing evidence that PCrL can act as a substitute for current surfactant technology by targeting lipid-based greasy stains.

#### 2.6.3. PCrL Compatibility with Laundry Detergents

**Figure 9:**
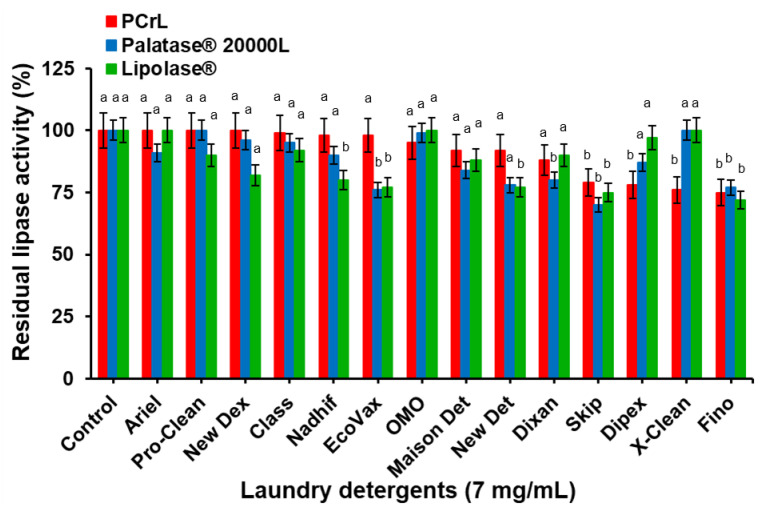
Stability and compatibility of the tested lipases in the presence of commercial laundry detergents. Lipases were incubated with laundry detergents (7 mg/mL) for 60 min at 37 °C. The enzyme activity of the control sample, without additive and incubated under similar conditions, was taken as 100%. Each point represents the mean of three independent experiments. Vertical bars indicate the standard error of the mean (*n* = 3). ^a–b^ Means in indicator enzymes with different lowercase letters differed significantly (*p* < 0.05).

To investigate the potential usage of PCrL in combination with commercially available liquid and solid detergents, the effects of these detergents on the stability of PCrL were observed and compared with those of Palatase^®^ 20000 L and Lipolase^®^. The lipase was pre-incubated in the presence of various commercial laundry detergents for 60 min at room temperature and the residual activity was determined under standard assay conditions, with TC8 as the substrate. As shown in Figure 9, the PCrL was remarkably stable and compatible with all of the commercial detergents tested, maintaining 75 to 100% of its original activity vs. 72 to 100% and 70 to 100% for Lipolase^®^ and Palatase^®^ 20000 L, respectively. PCrL showed, in particular, excellent compatibility and stability with Arial, Pro-Clean, New Dex, Class, Nadhif, and EcoVax, retaining 100% of its activity. Furthermore, Palatase^®^ 20000 L and Lipolase^®^ were shown to be less stable than PCrL in the presence of Nadhif, EcoVax, and New Det (Figure 9). All of these results also support the fact that PCrL possesses a number of encouraging properties that could provide new perspectives for laundry detergent preparations.

#### 2.6.4. Wash Performance Test on Oil Removal with PCrL

**Figure 10:**
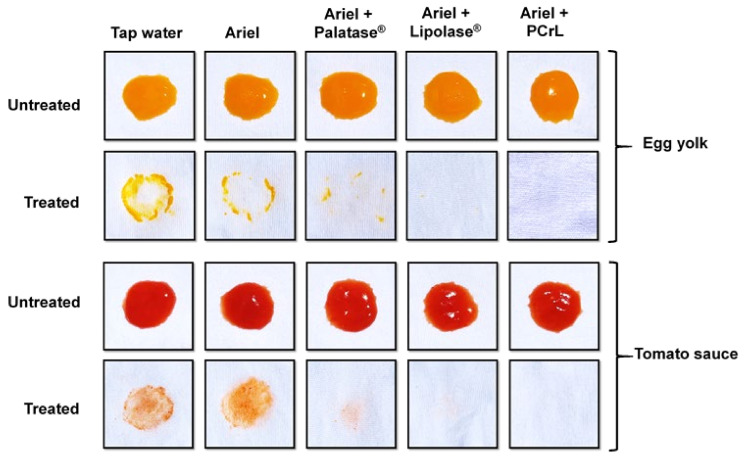
Wash performance test on oil removal with a commercial detergent in the presence of PCrL, Palatase^®^ 20000 L, or Lipolase^®^. The washing performance analysis test of the lipase was conducted with the commercial detergent Ariel (7 mg/mL) using cotton cloth stained with egg yolk or tomato sauce. The oil-stained cloth was rinsed with tap water, washed with Ariel (7 mg/mL), washed with Ariel supplemented with Palatase^®^ 20000 L (500 U/mL), washed with Ariel supplemented with Lipolase^®^ (500 U/mL), or washed with Ariel supplemented with PCrL (500 U/mL).

The effect of different commercial detergents on oil removal is shown in Table 6. Irrespective of the detergent used, the percentage of oil removal with a detergent added together with a lipase solution was found to be higher than that obtained with a detergent solution alone, which shows the advantage of including a lipase in the detergent formulation. Among all of the detergents (7 mg/mL) tested, the highest level of oil removal was observed with the Ariel detergent, as shown in Table 6. The addition of PCrL lipase to the detergent improved its efficacy by 46 to 100%, compared with 41 to 100% and 39 to 100% for Palatase^®^ 20000 L and Lipolase^®^, respectively (Table 6). The application of the lipase-free detergent resulted in less oil removal, with a decrease of between 21 and 54% (Table 6). These results were confirmed by the visual examination of the oily stains on cotton cloth stained with egg yolk or tomato sauce (Figure 10). Supplementing the detergent with PCrL, Palatase^®^ 20000 L, or Lipolase^®^ significantly improved the oil cleansing. In addition, PCrL facilitated the release of fatty acids in a much more efficient manner than Lipolase^®^, which is currently used. Furthermore, the results obtained during this study of the washing performance for the removal of oily stains from cotton cloths confirmed the potential of this lipase as a detergent additive.

## 3. Discussion

The morphological observation of strain P22, as well as the molecular approach, indicated that it was *Penicillium crustosum*. The *ITS* is the recommended DNA barcode for fungi [30]; however, this marker is not variable enough for distinguishing between all of the closely related species of *Penicillium* [31,32]. For that reason, β-tubulin is a better discriminative marker that allows the identification of *Penicillium* to species level [32,33]. The Bootstrap analysis revealed that the node values ranged from 50 to 99% in all groups, demonstrating their robustness, as reported elsewhere [34,35,36]. The yield of the PCrL purification procedure in just two steps was shown to be relatively high compared with that obtained in previous studies [15,37,38], which involved multiple steps of lipase purification from other *Penicillium* species. The SDS-PAGE results strongly suggest that PCrL is a monomeric protein comparable to those previously reported for other lipases from *Penicillium* strains. The molecular mass of PCrL (28 kDa) is similar to that of the *Penicillium expansum* strain PED-03 [39]. Most of the known lipases from the genus *Penicillium* have been reported to have a molecular mass in the range of 25–43 kDa [22,40,41].

The optimum temperature found for PCrL (37 °C) was seen to concur with the results for other *Penicillium* species, such as lipases from *Penicillium crustosum* strain 74 F [24] and other fungi of the genus *Penicillium*, for which the highest activities occurred between 25 and 45 °C [18]. For example, a temperature of 40 °C for maximal activity has been reported for lipase from *Penicillium cyclopium* [42]. It has been reported in the literature that the optimum temperature range for the lipase obtained from *Penicillium* species is between 25 and 60 °C [22]. The results of the effect of temperature on PCrL stability are similar to those reported for *Penicillium cyclopium* strain PG37, which is stable between 20 and 35 °C [43]. Thermal stability results have been reported for lipase from *Penicillium expansum* [44] and *Penicillium aurantiogriseum* [45], which exhibited relatively high temperature sensitivity and were unstable when incubated for 1 h at temperatures above 30 and 40 °C, respectively.

Lipases produced by the genus *Penicillium* have been reported to have optimum activity at neutral or basic pH values (ranging from 7 to 9) [45,46]; therefore, the optimum pH value of PCrL (pH 9) obtained in the present study appears to fit this pH pattern. An optimum pH of 9 for lipase activity has been reported for *Penicillium crustosum* strain 74 F [24] and *Penicillium notatum* [37]. The results of the high stability of PCrL at pH values ranging from 6 to 9 were similar to those reported for the lipase from *Penicillium crustosum* strain 74 F, with stability between pH 4 and 10 [24]. The high activity and stability at alkaline pH (see Figure 4) make PCrL a suitable biocatalyst for application in the detergent industry. Such enzymes that are active and stable at pH 8–10 have been incorporated into heavy-duty laundry and dishwashing detergents [47,48].

Orlistat, a known potent digestive lipase inhibitor, binds covalently to the active site serine of lipases [27]. PCrL was completely inhibited by Orlistat, suggesting the formation of a covalent complex between the β-lactone ring of Orlistat and the hydroxyl of the catalytic seryl residue of PCrL, as has been demonstrated for digestive lipases [27]. Additionally, PMSF and DFP were shown to completely inhibit PCrL activity, providing further evidence that the activity was associated with the catalytic Ser–His–Asp/Glu triad at the active site.

The relative stability of PCrL in the presence of chelating agents is a considerable advantage for the use of this enzyme as an additive in detergents. This is because commercial detergents contain large amounts of chelating agents, which work as water softeners and also help remove stains [49]. Increased PCrL activity with Ca^2+^ and Mn^2+^ has been reported for other *Penicillium* lipases [15,50]. In this group of enzymes, ions often play a structural role, rather than catalytic. It has been suggested that the metal ions induce a conformational change, thus promoting greater stability and, consequently, greater activity [51]. The inhibition of PCrL activity by Zn^2+^, Cu^2+^, Fe^2+^, and Ni^2+^ has been reported for other *Penicillium* lipases [15,52,53]. PCrL activity was completely inhibited by Hg^2+^ and Cd^2+^, as was reported for the lipase from *Haloferax mediterranei* [54]. The inhibition of many lipases by metal ions has most often been attributed to the reaction of metal ions with the thiol groups of cysteine residues near the active site of the enzyme [55].

In fact, various crystallographic data of microbial lipases show a conserved Ca^2+^ binding site close to the residues of the catalytic site, which would play a role in the stabilization of the overall structure of the protein and would contribute to the correct positioning of the residues of the catalytic site [56].

The results of the effect of NaTDC on the ability of PCrL to hydrolyze TGs are similar to those obtained with Lipolase^®^; it seems that PCrL is resistant to interfacial denaturation, since its activity on TC8 did not require any detergent. It was shown that Lipolase^®^ was able to bind TC8 emulsions in the absence of bile salts, but it is strongly desorbed from the interface in the presence of 4 mM NaTDC [57]. The inhibition exerted on PCrL by NaTDC at concentrations greater than 3 mM may be due to the fact that NaTDC, at its critical micellar concentration, prevents the binding of the enzyme at the lipid–substrate interface.

PCrL preferentially hydrolyzes medium-chain fatty acid esters, as reported for most *Penicillium* lipases, such as those of *Penicillium camembertii* Thom PG-3 [38], *Penicillium* *aurantiogriseum* [17], *Penicillium* *cyclopium* [42], and *Penicillium* *simplicissimum* [58]. Using tailor-made TGs, we demonstrated that PCrL is an *sn*-1,3-specific lipase with a clear regioselectivity toward the *sn*-1 position of the surface-coated TGs. These characteristics make PCrL a good catalyst for application in transesterification reactions involving the *sn*-1 position of the TG. Microbial lipases, through their regioselectivity, are of major importance and could be used for the modification of fatty substances and the production of structured dietary lipids, such as the production of cocoa butter substitutes or the enrichment of oils with unsaturated fatty acids through lipase-catalyzed interesterification and transesterification reactions [59]. For example, TLL has been used for the synthesis of 1,3-dioleoyl-2-palmitoylglycerol, a structured TG that could replace human milk fat in infant formula [60]. This TG, rich in palmitic acid in the *sn*-2 position and oleic acid in the *sn*-1,3 positions, was also synthesized by acidolysis and catalyzed by a commercial lipase from *Rhizopus oryzae* [61].

Organic solvents are widely used in enzymatic reactions with lipases because they facilitate the manipulation of hydrophobic substrates, such as lipids, and they can also modulate and/or increase the activity and selectivity of certain lipases [62]. In addition, lipases are widely exploited for their ability to catalyze the synthesis of new esters in aqueous–organic solvent mixtures or in pure organic solvents [7,18]. In this context, purified PCrL showed good stability in water-immiscible organic solvents, with residual activities of 94–150% after 24 h of incubation. Residual activities of 70–120% have been reported for the *Bjerkandera adusta* R59 lipase after 24 h of incubation at room temperature in cyclohexane, *n*-hexane, and *n*-heptane [63]. The stability of lipases in water-immiscible organic solvents has generally been attributed to the fact that they do not remove the hydration layer from the surface of the enzyme, which is necessary for catalytic activity [64]. In contrast, water-miscible hydrophilic solvents are incompatible with the activity of PCrL. Similarly, other lipases from *Penicillium* spp. showed significant instability in polar solvents [18,65]. The significant deactivation of enzymes in polar solvents may be due to the stripping-off of the essential bound water monolayer from the enzyme molecule [64]. Enzyme deactivation in media containing organic solvents is most likely also caused by the disruption of the hydrophobic core of the protein molecule due to the change in the hydrophobicity of the medium [66]. In particular, polar solvents (such as acetonitrile and ethyl acetate), which can penetrate the protein, are able to induce more structural changes for the interaction between the active site and substrate than non-polar solvents, as previously reported by Serdakowski and Dordick [67].

Laundry detergents are a mixture of large components, such as bleaching agents, surfactants, enzymes, and builders, preventing the deposition of soil onto fabrics, preventing corrosion, adding alkalinity, and retaining buffering capacity [68]. Bleaching agents provide the bleaching action through oxidation, whilst the surfactants decrease the surface tension at the interfaces, thus improving the repulsive force and promoting the cleaning process. The role of the enzymes is to make the cleaning procedure more efficient. This is why alkaline biocatalysts, such as PCrL, must be compatible with all of the usual constituents of detergents. In fact, the stains are removed by mechanical action assisted by the catalytic action of enzymes. The use of biocatalysts in laundry detergents constitutes the largest segment of the global industrial enzyme market. The main enzymes in biological laundry detergents are proteases, but it has become more common recently to incorporate a mixture of enzymes, including lipases, amylases, mananases, and peroxidases.

## 4. Material and Methods

### 4.1. Material

Rhodamine B, NaTDC, DMSO, bovine serum albumin (BSA), β-cyclodextrin (β-CD), gum arabic (GA), polyvinylidene difluoride (PVDF) transfer membrane, phenylmethanesulfonyl fluoride (PMSF), diiodopropyl fluorophosphate (DFP), and chemical reagents were obtained from Sigma-Aldrich Chimie (Saint-Quentin-Fallavier, France). DTNB, NEM, iodoacetamide, PAO, and chemical reagents were also obtained from Sigma-Aldrich Chimie (Saint-Quentin-Fallavier, France). Orlistat (tetrahydrolipstatin), a known digestive lipase inhibitor, was obtained from Hoffmann-La-Roche Ltd (Basel, Switzerland), Lipolase^®^, a 1,3 specific lipase from *Thermomyces lanuginosus*, and Palatase^®^ 20000 L, a 1,3 specific lipase originating from *Rhizomucor miehei*, were supplied by Novozymes Biopharma DK A/S (Bagsvaerd, Denmark). The ÄKTA prime FPLC system was equipped with a HiTrap™ Q-Sepharose Fast Flow (FF) column (GE Healthcare, Bio-Sciences AB, Uppsala, Sweden) with a 5 mL volume.

### 4.2. Lipids

TC2, TC4, TC6, TC8, OO were obtained from Sigma-Aldrich Chimie (Saint-Quentin-Fallavier, France). Synthetic TGs substrates were esterified with α-eleostearic acid (9*Z*,11*E*,13*E*-octadecatrienoic acid) at either the *sn*-1 position (*sn*-EOO) or at the *sn*-3 position (*sn*-OOE) by laboratory synthesis according to the protocol described previously [28]. α-eleostearic acid, with its conjugated triene, constitutes an intrinsic chromophore and, therefore, confers the strong ultraviolet (UV) absorption properties of this free fatty acid, as well as those of the TG esterified by it. A non-hydrolyzable ether linkage with a non-UV absorbing alkyl chain was introduced at the other *sn* positions to prevent the migration of the acyl chain during TG lipolysis [28] (the structures of *sn*-EOO and *sn*-OOE are shown in the insert of Figure 7).

### 4.3. Isolation of a Lipase-Producing Strain, Media, and Culture Conditions

The fungal strain P22 used in this study was isolated from Moroccan OMW. The qualitative screening for lipase activity was performed using the plate assay described by Abdullah et al. [69]. The isolate was inoculated on the solid culture medium potato dextrose agar (PDA) supplemented, after autoclaving, with 1% (*v*/*v*) of OO and 0.01% (*w*/*v*) of rhodamine B. After 4 days of incubation at 25 °C, the lipase activity was detected by irradiating the plates with UV light at 350 nm. The lipase activity was detected by the appearance of an orange fluorescent halo around the colonies. The strain P22 was then cultured on a PDA medium, incubated at 25 °C for 4 days, maintained at 4 °C when spores were formed, and subcultured every 2 weeks.

### 4.4. Taxonomic Identification of Fungal Strain P22

The isolate P22 was identified based on macroscopic and microscopic morphological characterizations, as described by Visagie et al. [33]. It was also identified at a molecular level at the Westerdijk Fungal Biodiversity Institute (CBS, Utrecht, the Netherlands) according to the following protocol: the DNA was extracted using a Qiagen DNeasy Ultraclean™ Microbial DNA Isolation Kit. Fragments containing the Internal Transcribed Spacer (*ITS*) 1 and 2 regions. including the 5.8S rDNA (*ITS*), a partial β-tubulin gene (*benA*), and fragments containing a partial calmodulin gene (*cam*) were amplified and sequenced. PCR amplification was performed using Taq DNA polymerase with aliquots of each cell lysate suspension or genomic DNA in Tris-EDTA buffer as a template. The PCR amplification consisted of an initial denaturation for 5 min at 94 °C, followed by 35 cycles, each consisting of 45 s of denaturation at 94 °C, annealing for 45 s at 55 °C, and extension for 1 min (according to the size of the region examined) at 72 °C. The final extension step was carried out at 72 °C for 7 min. PCR products were analyzed on 1% agarose gel and visualized with specific DNA dyes. The primers used were: *ITS*: LS266 (GCATTCCCAAACAACTCGACTC) and V9G (TTACGTCCCTGCCCTTTGTA); *benA*: BT2a (GGTAACCAAATCGGTGCTGCTTTC) and BT2b (ACCCTCAGTGTAGTGACCCTTGGC); and *cam*: CMD5 (CCGAGTACAAGGARGCCTTC) and CMD6 (CCGATRGAGGTCATRACGTGG). The PCR fragments were sequenced in both directions with the primers used for PCR amplification using an ABI Prism^®^ Big DyeTM Terminator v. 3.0 Ready Reaction Cycle Sequencing Kit. Samples were analyzed on an ABI PRISM 3700 Genetic Analyzer, and contigs were assembled using the forward and reverse sequences with the program SeqMan from the LaserGene package. The sequences were compared on GenBank using BLAST and in the in-house sequence database of the Westerdijk Fungal Biodiversity Institute. Phylogenetic and molecular evolutionary genetic analyses were performed using the Molecular Evolutionary Genetics Analysis (MEGA) software v. 4.1. Distances and clustering were calculated using the neighbor-joining method. The tree topology of the neighbor-joining data was evaluated by Bootstrap analysis with 100 re-samplings.

### 4.5. Lipase Activity Measurement

Lipase activity was continuously assayed potentiometrically by measuring the free fatty acids (FFAs) released from mechanically stirred TG emulsion using 0.1 M NaOH and a pH-Stat device (Metrohm 702 SM Titrino, Herisau, Switzerland). Each assay was performed in a thermostat (37 °C) vessel containing 0.5 mL of TG substrate and 14.5 mL of 0.3 mM Tris-HCl buffer (pH 9) containing 150 mM of NaCl and 2 mM of CaCl_2_. When OO was used as a substrate, it was first pre-emulsified with GA by mixing 10 mL of OO with 90 mL of a 10% (*w*/*v*) GA solution, prepared as described elsewhere [70]. The OO emulsion (5 mL) was then mixed in the pH-Stat vessel with 10 mL of the assay buffer. The enzymatic activity was expressed in international units, (1 IU) corresponding to 1 μmol of FFA released per min.

A continuous spectrophotometric assay was conducted to determine the regioselectivity of the PCrL toward TGs using surface-coated *sn*-EOO or *sn*-OOE as substrates [28]. The wells of microtiter plates were coated with synthetic TGs as described in our previous report [28]. The TG-coated wells in the microtiter plate were first washed three times with Tris-HCl buffer (10 mM, pH 8.0) containing 6 mM CaCl_2_, 150 mM NaCl, 1 mM EDTA, and 2.5 mM β-CD, and left to equilibrate with the reaction buffer (200 µL) for 10 min at 37 °C. The PCrL (0.65 μg) was then injected into the microtiter plate wells and the absorbance at 272 nm was recorded at regular 1 min intervals via a microplate spectrophotometer (Tecan Infinite M200 Pro), with 5 s of shaking of the microtiter plate before each reading. Absorbance values recorded at 272 nm were compared with the buffer alone as a control. The specific activity of PCrL, expressed as the variation in the absorbance per minute, was determined using α-eleostearic acid with an apparent molar extinction of 5320 M^−1^ cm^−1^ [28]. The following equation was used to calculate the RI% of PCrL toward TGs:

(1) $$\mathrm{RI}=\frac{\left({\;A}_{\mathrm{sn}-\mathrm{EOO}}-A_{\mathrm{sn}-\mathrm{OOE}}\;\right)}{A_{\mathrm{sn}-\mathrm{EOO}}+{\;A}_{\mathrm{sn}-\mathrm{OOE}}}\times 100$$

where A*_sn_*_-EOO_ and A*_sn_*_-OOE_ are the specific activities of PCrL using *sn*-EOO and *sn*-OOE, respectively, as substrates.

### 4.6. Lipase Production

The liquid medium used for lipase production was prepared as described by Dheeman et al. [15], with minor modifications, and consisted of (g/L): peptone casein (15), yeast extract (5), KH_2_PO_4_ (1.75), MgSO_4_ (0.5), and 1% (*v*/*v*) OO at pH 7. For the preparation of the inoculum, 0.5 mL of spore suspension (10^5^ spores/mL) of the strain P22 was inoculated into 50 mL of the medium in a 250 mL Erlenmeyer flask and incubated with shaking (120 rpm and 25 °C). Ten-day-old cultures were used for the lipase assay and biomass quantification.

### 4.7. Analytical Methods

The protein concentration was determined as described by Bradford [71] using Bio-Rad Dye Reagent and BSA as the standard. The active lipase fractions were analyzed by polyacrylamide gel (12%) electrophoresis in the presence of sodium dodecyl sulfate (SDS-PAGE) and β-mercaptoethanol (β-ME) according to the Laemmli method [72].

After SDS-PAGE analysis, the purified lipase was electrotransferred onto a PVDF membrane using the Mini Trans-Blot electrophoretic transfer cell Bio-Rad according to the manufacturer’s instructions. The PVDF membrane was rinsed with distilled water and stained with Ponceau red. The NH_2_-terminal amino-acid sequence of the protein-bearing band was determined by automated Edman degradation using a PPSQ-31B protein sequencer from Shimadzu (Kyoto, Japan).

### 4.8. Purification Procedure

After a 6-day culture of the strain P22, when the lipase activity was maximal, the mycelium was filtered on Whatman filter paper grade No. 1, size 110 mm, and the crude enzyme was precipitated with ammonium sulfate at a concentration of 70% saturation. Proteins were collected by centrifugation at 12,000× *g* for 20 min and 4 °C, resuspended in an appropriate volume of 50 mM Tris-HCl at pH 8, supplemented with 200 mM NaCl and 2 mM benzamidine, and dialyzed overnight against 50 mM Tris-HCl at pH 8 and 2 mM benzamidine (buffer A) at 4 °C. Anion exchange chromatography was carried out on an ÄKTA prime FPLC system equipped with a HiTrap™ Q-Sepharose FF column (5 mL), pre-equilibrated with buffer A. The adsorbed proteins were eluted with a linear gradient of NaCl (0–200 mM) in buffer A at a flow rate of 1 mL/min. Chromatographic fractions containing lipase activity were pooled and concentrated using a 10 kDa centrifugal concentrator (Merck Amicon^®^, Beverly Hills, CA, USA).

### 4.9. Biochemical Characterization of the Purified PCrL

#### 4.9.1. Effects of Temperature on Lipase Activity and Stability

The optimum temperature of PCrL was determined using the pH-Stat technique as described above (Section 4.5) at pH 9 and at a temperature range varying from 20 to 50 °C using TC8 as the substrate. To study the thermal stability, PCrL was incubated at temperatures ranging from 20 to 50 °C for 1 h, and residual PCrL activity was measured under standard assay conditions (pH 9 and 37 °C).

#### 4.9.2. Effects of pH on Lipase Activity and Stability

The optimum pH of PCrL was determined using the pH-Stat technique as described above (Section 4.5) at a pH ranging from 5 to 11 at 37 °C. The effect of pH on lipase stability was determined by incubating PCrL at different pH values, ranging from 5 to 11, for 1 h at 4 °C using different buffers at 50 mM: citrate (pH 5–6), potassium phosphate (pH: 6–7), Tris-HCl (pH: 7–9), and glycine-NaOH (pH: 9–11). The residual PCrL activity was measured under standard assay conditions.

#### 4.9.3. Substrate Specificity

The substrate specificity profile of PCrL was studied by using TG substrates with different chain lengths: TC2, TC4, TC6, TC8, and OO. The enzymatic activities were determined on each substrate by the titrimetric method.

#### 4.9.4. Effects of Inhibitors, Reducing Agents, Chelating Reagents, Metal Ions, and Bile Salts on PCrL Activity

The effects of Orlistat, PMSF, DFP, benzamidine hydrochloride hydrate, DTNB, NEM, iodoacetamide, PAO, EDTA, EGTA, β-ME, and dl-DTT, as well as various metal ions (2 mM) (Ca^2+^, Mn^2+^, Mg^2+^, Fe^2+^, Cu^2+^, Zn^2+^, and Co^2+^), on lipase stability were all investigated by pre-incubating the purified PCrL enzyme for 1 h at 37 °C with each inhibitor or reducing agent, or in the presence of each metallic ion. Enzyme assays were carried out under standard assay conditions. Orlistat (53 µM diluted in DMSO) was injected a few minutes after lipase addition and the PCrL activity was then continuously recorded.

In order to study the effect of bile salt on PCrL activity, the enzyme was measured by the pH-Stat technique under standard assay conditions in the presence of increasing concentrations of NaTDC (from 1 to 10 mM). PCrL was injected into the reaction vessel of a pH-Stat containing the emulsified TC8 substrate under vigorous stirring.

### 4.10. Performance Evaluation of the Purified PCrL Compared with Palatase^®^ 20000 L and Lipolase^®^

#### 4.10.1. Effect of Organic Solvents on Lipase Stability

The organic solvent stability of PCrL was studied by incubating the enzyme preparation with various organic solvents and different logarithms of the partition coefficient (Log *P*) values at 25% (*v*/*v*) for 24 h at 37 °C with constant shaking of 200 rpm. Log *P* is a quantitative measure of solvent polarity that can be useful in determining the stability of enzymes in organic solvents [73]. Aliquots were taken at the requisite time intervals to test the remaining activity. The reaction mixture without any additives was taken as a control (100%). The organic solvents used were classified according to their Log *P* values: *n*-hexadecane (8.8), *n*-decane (5.6), *iso*-octane (4.5), *n*-hexane (3.5), cyclohexane (3.3), toluene (2.5), chloroform (1.97), *n*-hexanol (1.8), *n*-butanol (0.88), ethyl acetate (0.73), *iso*-propanol (0.28), acetonitrile (−0.15), ethanol (−0.24), methanol (−0.76), dimethylformamide (DMF, −1.03), and DMSO (−1.35).

#### 4.10.2. Effect of Some Detergent Additives on PCrL Stability

The stability of PCrL in several laboratory-available detergent additives and in known commercial detergents was determined after incubating PCrL for 1 h at 37 °C. The detergent additives used in this work were as described recently [34]: zeolite, STPP, SDS, Na_2_ CMC, Na_2_CO_3_, Tween (20, 40, 60, and 80), Triton X100, TAED, NaBO_3_, H_2_O_2_, SAFOL 23E7, Dehydol^®^ LT 7, SURFAC^®^ LM 30, NEODOL^®^ 25-7, Galaxy LAS, Galaxy LES 70, Galaxy 110, Galaxy CAPB Plus, TERGITOLTM NP-9, SURFACTANT, FINDET^®^ AR/52, anti-foam, formol, Tinopal^®^ CBS-X, Sulfacid K, Marlipal^®^ 31/90, EDTA, Class Perfumes I and II, Propyl betaine, and NaOH 50%.

The compatibility of the PCrL with some solid and liquid laundry detergents was determined as detailed previously [68]. Briefly, each commercial laundry detergent was dissolved in tap water to reach a concentration of 7 mg/mL and treated for 1 h at 65 °C to deactivate endogenous lipases. After that, PCrL, at 500 U/mL, was added and incubated with shaking for 1 h at 37 °C with each commercial detergent. The residual PCrL activity was measured under standard assay conditions.

#### 4.10.3. Washing Performance Analysis of PCrL

The washing performance of PCrL as a bio-detergent additive was evaluated on pieces of white cotton cloth (6 × 6 cm) that had been stained with oily egg yolk or tomato sauce. The stained fabric pieces were incubated in different wash treatments at 37 °C and agitated at 200 rpm for 30 min in 250 mL Erlenmeyer flasks containing a total volume of 100 mL of tap water, Ariel detergent at a final concentration of 7 mg/mL (heat-inactivated), and supplemented with PCrL solution (at 500 U/mL). After that, the fabric pieces were removed, rinsed with distilled water, dried, and subjected to visual observation to test the stain removal effects of lipase. The OO was extracted with petroleum ether for 6 h using a Soxhlet extractor after the complete evaporation of petroleum ether from the extract. The percentage of OO removed was calculated using the following equation [74]:

Removal (%) = [Weight of total OO before washing (mg) − Weight of total OO after washing (mg)]/[Weight of total OO after washing (mg)] × 100

### 4.11. Statistical Analysis

All experiments were performed with at least three independent replicates, and the control without lipase was analyzed under identical standard experimental conditions. The data determinations are expressed as the mean of the replicate determinations and standard deviation (±SD). The Student–Newman–Keuls (S-N-K) comparison test was used to identify the group of means by one-way analysis of variance (ANOVA), followed by means comparisons. A *p* value of <0.05 was considered statistically significant. Statistical analysis was carried out using Statgraphics 18.1.08 software.

### 4.12. Culture Collection Depository’s Numbers and Nucleotide Sequence Accession Numbers

The Actinomycota fungus culture of the P22 isolate was deposited in the Moroccan Coordinated Collections of Microorganisms (CCMM) in accordance with the Rules of the Bacteriological Code (1990 revision) as revised by the ICSP at the plenary sessions in Sydney and in Paris at the “Centre National pour la Recherche Scientifique et Technique (CNRST)”, Rabat (Morocco), and at the Westerdijk Fungal Biodiversity Institute (WFDI), Royal Netherlands Academy of Arts and Sciences (KNAW), Uppsalalaan, Utrecht (the Netherlands) under the authentic culture numbers: CCMM M185 and CBS: 21.150/Det, respectively. The nucleotide sequences of the 5.8S rRNA, *benA*, and *cmd* genes reported in this study were submitted to the GenBank/ENA/DDBJ databases under the accession numbers ON854673, ON854674, and ON854675, respectively.

## 5. Conclusions

In the present study, the extracellular alkaline lipase produced by *Penicillium crustosum* Thom strain P22 was proven to have numerous novel properties of prominent industrial value, particularly an alkaline pH and temperature stability. Compared with Palatase^®^ and Lipolase^®^, PCrL showed considerable catalytic effectiveness, an elevated tolerance to organic solvents, as well as exceptional stability and compatibility with a wide range of commercialized laundry detergents. Overall, the findings indicate that PCrL has promising properties that could lead to its use in laundry detergent formulations. Considering the attractive properties and attributes of the PCrL enzyme, additional studies are needed to reveal the molecular structure of its encoding gene *lipCrL* and its regulatory region, and to examine its structure–functions relationship using site-directed mutagenesis (SMD) and 3D structural modeling.

## Figures and Tables

**Table 1:** Flow sheet of the PCrL purification steps.

Purification Steps ^a^	Total Activity (U)	Total Protein (mg)	Specific Activity (U/mg)	Purification (-Fold)	Yield (%)
Crude extract	7680 ± 21	48.0 ± 1	160 ± 2.5	1	100
(NH_4_)_2_SO_4_ precipitation (70%)	3900 ± 10	15.3 ± 0.5	254 ± 4.0	1.6	51
HiTrap Q-Sepharose FF column	2600 ± 6	0.2 ± 0	10,000 ± 5	62.5	34

^a^ Experiments were conducted with 160 mL of 6-day-old culture medium at least three times independently and the standard errors are shown.

**Table 2:** Alignment of the NH_2_-terminal amino-acid sequence of PCrL with that of other *Penicillium* lipases.

Origin	NH_2_-Terminal Amino Acid ^a^	Identity (%)
*Penicillium crustosum* strain P22 (this study)	ATADAAAFLDLHMAAKLSSA ^b^	-
*Penicillium cyclopium* strain PG37 (AF274320)	ATADAAAFPDLHRAAKLSSA	90
*Penicillium camemberti* strain FM013 (CRL22273)	ATAATAAFPDLHRAAKLSSA	80
*Penicillium solitum* strain IBT 29525 (XP_040813639)	ATAATAAFPDLNXAAKLSSA	75
*Penicillium expansum* strain DSM 1994 (AAB27002)	AVAASAAFPDLXRAAKLSSA	70

^a^ Residues not identical with the PCrL are shaded. The GenBank accession number is in parentheses. ^b^ Amino acid sequences for comparison were obtained using the program BLASTP (NCBI, NIH, USA) database. X: any amino acid.

**Table 3:** Effects of various inhibitors, chemical reagents, and metallic ions on the purified lipase PCrL. The enzyme assay was performed after the pre-incubation of the enzyme with each inhibitor or reducing agent, or each metallic ion-tested compound, for 1 h at 37 °C. The lipase activity measured in the absence of any inhibitor or reducing agent was taken as the control (100%). The non-treated and dialyzed enzyme was considered as 100% for the metal ion assay. Residual activity was measured at pH 9 and 37 °C.

Inhibitors/Chemical Reagents/Metal Ions ^a^	Concentration ^a^	Residual Lipase Activity (%) ^b^
None	-	100 ^b^ ± 3
PMSF	5 mM	0
DFP	2 mM	0
Benzamidine	2 mM	110 ^b^ ± 3
DTNB	10 mM	73 ^bc^ ± 2
NEM	2 mM	95 ^b^ ± 2
Iodoacetamide	5 mM	106 ^b^ ± 3
PAO	10 mM	94 ^b^ ± 2
β-ME	10 mM	96 ^b^ ± 2
dl-DTT	10 mM	98 ^b^ ± 2
EDTA	10 mM	92 ^b^ ± 2
EGTA	1 mM	96 ^b^ ± 2
Ca^2+^ (CaCl_2_)	2 mM	147 ^a^ ± 4
Mn^2+^ (MnCl_2_)	2 mM	146 ^a^ ± 4
Mg^2+^ (MgCl_2_)	2 mM	92 ^b^ ± 2
Fe^2+^ (FeSO_4_)	2 mM	70 ^bc^ ± 2
Co^2+^ (CoCl_2_)	2 mM	41 ^cd^ ± 1
Ba^2+^ (BaCl_2_)	2 mM	96 ^b^ ± 2
Zn^2+^ (ZnSO_4_)	2 mM	9 ^d^ ± 0
Cu^2+^ (CuCl_2_)	2 mM	23 ^d^ ± 1
Ni^2+^ (NiCl_2_)	2 mM	16 ^d^ ± 0
Hg^2+^ (HgCl_2_)	2 mM	0
Cd^2+^ (CdCl_2_)	2 mM	0

^a–d^ Means in the same column of each parameter with different lower-case letters differed significantly (*p* < 0.05). Incubation with the purified enzyme at 37 °C for 1 h. Values represent the means of three independent replicates, and ± SEs are shown.

**Table 4:** Stability of alkaline lipases PCrL, Palatase^®^ 20000 L, and Lipolase^®^ with several detergent additives.

Detergent Additives	Final Concentration	Residual Lipase Activity (%) ^a,b^		
		PCrL	Palatase^®^ 20000 L	Lipolase^®^
Control	0	100 ^cd^ ± 3	100 ^cd^ ± 3	100 ^cd^ ± 3
Zeolite	1% (*w*/*v*)	95 ^cd^ ± 2	91 ^cd^ ± 2	86 ^cd^ ± 2
STPP	0.2% (*w*/*v*)	150 ^ab^ ± 4	146 ^ab^ ± 4	15 ^f^ ± 4
	0.5% (*w*/*v*)	98 ^cd^ ± 2	87 ^cd^ ± 2	97 ^cd^ ± 2
SDS	1% (*w*/*v*)	185 ^a^ ± 4	90 ^cd^ ± 2	96 ^cd^ ± 2
	2% (*w*/*v*)	150 ^ab^ ± 4	66 ^de^ ± 2	75 ^d^ ± 2
Na_2_CO_3_	50 mM	171 ^a^ ± 4	165 ^a^ ± 4	154 ^ab^ ± 4
	100 mM	147 ^ab^ ± 4	141 ^ab^ ± 4	126 ^bc^ ± 3
Tween 20	1% (*v*/*v*)	96 ^cd^ ± 2	87 ^cd^ ± 2	73 ^d^ ± 2
	5% (*v*/*v*)	49 ^de^ ± 1	76 ^d^ ± 2	54 ^de^ ± 1
Tween 40	1% (*v*/*v*)	108 ^bc^ ± 3	98 ^cd^ ± 2	87 ^cd^ ± 2
	5% (*v*/*v*)	45 ^e^ ± 1	43 ^e^ ± 1	36 ^ef^ ± 1
Tween 60	1% (*v*/*v*)	84 ^cd^ ± 2	95 ^cd^ ± 2	98 ^cd^ ± 2
	5% (*v*/*v*)	31 ^ef^ ± 1	67 ^de^ ± 2	60 ^de^ ± 2
Tween 80	1% (*v*/*v*)	83 ^cd^ ± 2	93 ^cd^ ± 2	90 ^cd^ ± 2
	5% (*v*/*v*)	29 ^ef^ ± 1	52 ^de^ ± 1	50 ^de^ ± 1
Na_2_CMC	1% (*w*/*v*)	143 ^ab^ ± 4	96 ^cd^ ± 2	92 ^cd^ ± 2
	10% (*w*/*v*)	105 ^c^ ± 3	64 ^de^ ± 2	52 ^de^ ± 1
Triton X-100	1% (*v*/*v*)	140 ^ab^ ± 4	80 ^cd^ ± 2	85 ^cd^ ± 2
	5% (*v*/*v*)	103 ^cd^ ± 3	66 ^de^ ± 2	68 ^de^ ± 2
TAED	0.5% (*w*/*v*)	142 ^ab^ ± 4	126 ^bc^ ± 3	116 ^bc^ ± 3
	5% (*w*/*v*)	103 ^cd^ ± 3	92 ^cd^ ± 2	75 ^d^ ± 2
Sodium perborate	1% (*v*/*v*)	139 ^ab^ ± 3	80 ^b^ ± 2	113 ^bc^ ± 3
	5% (*v*/*v*)	99 ^cd^ ± 3	51 ^de^ ± 1	88 ^cd^ ± 2
H_2_O_2_	5% (*v*/*v*)	146 ^abA^ ± 4	61 ^deA^ ± 2	80 ^cdA^ ± 2
	10% (*v*/*v*)	104 ^cdA^ ± 3	45 ^eA^ ± 1	66 ^deA^ ± 2

^a–f^ Means in the same column of each parameter with different lower-case letters differed significantly (*p* < 0.05). The lipase was incubated for 1 h with a detergent additive at 37 °C, and the residual lipase activity was determined under optimal assay conditions for each enzyme. Data presented are the average of at least 3 sets of tests, ±SE. ^A^ Data collected in the presence of 100 mM borate-NaOH buffer.

**Table 5:** Stability of alkaline lipases PCrL, Palatase^®^ 20000 L, and Lipolase^®^ with several commercialized detergent additives.

Commercialized Detergent Additives	Concentration (%, *v*/*v*)	Residual Lipase Activity (%) ^a,b^		
		PCrL	Palatase^®^ 20000 L	Lipolase^®^
Control	0	100 ^cd^ ± 3	100 ^cd^ ± 3	100 ^cd^ ± 3
SAFOL 23 E7	0.5	152 ^ab^ ± 4	145 ^ab^ ± 4	142 ^ab^ ± 4
	1	95 ^cd^ ± 2.4	93 ^cd^ ± 2	90 ^cd^ ± 2
Dehydol^®^ LT 7	0.5	154 ^ab^ ± 4	142 ^ab^ ± 4	164 ^a^ ± 4
	1	99 ^cd^ ± 2	95 ^cd^ ± 2	108 ^bc^ ± 3
SURFAC^®^ LM 30	0.5	183 ^a^ ± 5	142 ^ab^ ± 4	120 ^bc^ ± 3
	1	150 ^ab^ ± 4	102 ^cd^ ± 3	94 ^cd^ ± 2
NEODOL^®^ 25-7	0.5	75 ^d^ ± 2	96 ^cd^ ± 2	87 ^cd^ ± 2
	1	25 ^ef^ ± 1	57 ^de^ ± 1	45 ^e^ ± 1
Galaxy LAS	2	82 ^cd^ ± 2	99 ^cd^ ± 2	95 ^cd^ ± 2
	5	30 ^ef^ ± 1	60 ^de^ ± 2	59 ^de^ ± 1
Galaxy LES 70	2	110 ^bc^ ± 3	103 ^cd^ ± 3	94 ^cd^ ± 2
	5	55 ^de^ ± 1	53 ^de^ ± 1	50 ^de^ ± 1
Galaxy 110	2	149 ^ab^ ± 4	150 ^ab^ ± 4	125 ^cb^ ± 3
	5	93 ^cd^ ± 2	98 ^cd^ ± 2	83 ^cd^ ± 2
Galaxy CAPB Plus	1	153 ^ab^ ± 4	162 ^a^ ± 4	14 ^f^ ± 4
	5	105 ^c^ ± 3	106 ^b^ ± 3	105 ^c^ ± 4
TERGITOL^TM^ NP-9 SURFACTANT	2	86 ^cd^ ± 2	76 ^d^ ± 2	89 ^cd^ ± 2
	5	30 ^ef^ ± 1	46 ^e^ ± 1	50 ^de^ ± 1
FINDET^®^ AR/52	0.5	78 ^d^ ± 2	82 ^cd^ ± 2	75 ^d^ ± 2
	1	26 ^ef^ ± 1	35 ^ef^ ± 1	28 ^ef^ ± 1
Anti-foam	0.5	85 ^cd^ ± 2	98 ^cd^ ± 2	88 ^cd^ ± 2
	1	46 ^e^ ± 1	56 ^de^ ± 1	50 ^ed^ ± 1
Formol	0.2	160 ^ab^ ± 4	153 ^ab^ ± 4	135 ^a^ ± 3
	0.5	108 ^b^^c^ ± 3	96 ^cd^ ± 2	84 ^cd^ ± 2
Tinopal^®^ CBS-X	0.5	152 ^ab^ ±4	164 ^a^ ± 4	122 ^bc^ ± 3
	1	95 ^cd^ ± 2	111 ^bc^ ± 3	86 ^cd^ ± 2
Sulfacid K	10	77 ^d^ ± 2	84 ^cd^ ± 2	83 ^cd^ ± 2
	15	15 ^f^ ± 1	32 ^ef^ ± 1	25 ^ef^ ± 1
Marlipal^®^ 31/90	0.5	79 ^cd^ ± 2	66 ^de^ ± 2	83 ^cd^ ± 2
	1	30 ^ef^ ± 1	42 ^ef^ ± 1	35 ^ef^ ± 1
EDTA	0.2	130 ^b^ ± 3	111 ^bc^ ± 3	125 ^bc^ ± 3
	0.5	76 ^d^ ± 2	71 ^de^ ± 2	62 ^de^ ± 2
Perfume I Class	0.5	165 ^ab^ ± 4	152 ^ab^ ± 4	146 ^ab^ ± 4
	1	109 ^bc^ ± 3	101 ^b^ ± 3	99 ^cd^ ± 2
Perfume II Class	0.5	170 ^a^ ± 4	162 ^a^ ± 4	155 ^ab^ ± 4
	1	110 ^bc^ ± 3	105 ^c^ ± 3	96 ^cd^ ± 2
Propyl betaine	1	95 ^cd^ ± 2	85 ^cd^ ± 2	81 ^cd^ ± 2
NaOH 50%	1	113 ^bc^ ± 3	122 ^bc^ ± 3	119 ^bc^ ± 3

^a–f^ Means in the same column of each parameter with different lower-case letters differed significantly (*p* < 0.05). The lipase was incubated for 1 h with detergent additives at 37 °C, and the residual activity was determined under optimal assay conditions for each enzyme. Data presented are the averages of at least 3 sets of tests, ±SE.

**Table 6:** Effect of PCrL, Palatase^®^ 20000 L, and Lipolase^®^ on the removal of olive oil from cotton fabric with various marketed liquid and solid laundry detergents.

Laundry Detergent (7 mg/mL)	Oil Removal (%)			
	Detergent	Detergent + PCrL	Detergent + Palatase^®^ 20000 L	Detergent + Lipolase^®^
Ariel	54 ^bc^ ± 2	100 ^a^ ± 3	93 ^a^ ± 2	98 ^a^ ± 3
OMO	40 ^c^ ± 1	81 ^a^ ± 2	88 ^a^ ± 2	100 ^a^ ± 3
Nadhif	34 ^c^ ± 1	51 ^bc^ ± 2	45 ^bc^ ± 1	42 ^c^ ± 1
EcoVax	35 ^c^ ± 1	72 ^b^ ± 1	53 ^bc^ ± 2	61 ^b^ ± 1
Dipex	27 ^cd^ ± 1	54 ^bc^ ± 1	59 ^b^ ± 2	68 ^b^ ± 1
Maison Det	44 ^c^ ± 1	91 ^a^ ± 2	76 ^ab^ ± 1	82 ^a^ ± 2
Pro-Clean	55 ^bc^ ± 1	100 ^a^ ± 3	100 ^a^ ± 3	87 ^a^ ± 2
New Det	29 ^c^ ± 1	61 ^b^ ± 1	41 ^c^ ± 1	39 ^c^ ± 1
New Dex	39 ^c^ ± 1	98 ^a^ ± 3	91 ^a^ ± 2	86 ^a^ ± 2
Class	49 ^bc^ ± 2	96 ^a^ ± 2	89 ^a^ ± 2	86 ^a^ ± 2
Fino	21 ^d^ ± 2	46 ^bc^ ± 1	50 ^cb^ ± 2	39 ^c^ ± 1
X-Clean	46 ^bc^ ± 1	90 ^a^ ± 2	100 ^a^ ± 3	98 ^a^ ± 3
Dixan	21 ^d^ ± 1	54 ^bc^ ± 1	45 ^cb^ ± 1	59 ^b^ ± 2
Skip	25 ^cd^ ± 1	65 ^b^ ± 1	57 ^b^ ± 1	62 ^b^ ± 1

^a–d^ Means in for all columns of each parameter with different lower-case letters differed significantly (*p* < 0.05). Values represent the means of 3 independent replicates and the ± SE is shown.

## Data Availability

The datasets generated and/or analyzed during the current study are available on the GenBank repository, https://www.ncbi.nlm.nih.gov/genbank/. The GenBank accession numbers for the nucleotide sequences of the 5.8S rRNA, *benA*, and *cmd* genes referred to in the text are ON854673, ON854674, and ON854675, respectively. Other datasets generated during and/or analyzed during the current study available from the corresponding authors on reasonable request.
